# Nutrition transition, double burden of malnutrition, and urbanization patterns in secondary cities of Bangladesh, Kenya and Rwanda

**DOI:** 10.1186/s40795-023-00782-1

**Published:** 2023-11-04

**Authors:** Tanja Barth-Jaeggi, Cornelia Speich, Cassien Havugimana, Francine Bayisenge, Simon Kimenju, Wilfred Omondi, S. Fuad Pasha, Shahidul Islam, Kesso Gabrielle van Zutphen-Küffer, Sophie van den Berg, Dominique Barjolle, Marnie Pannatier, Helen Prytherch

**Affiliations:** 1 Swiss Tropical and Public Health Institute, Allschwil, Switzerland; 2 University of Basel, Basel, Switzerland; 3 Swiss TPH, Kigali Office, Kigali, Rwanda; 4 Kula Vyema Centre of Food Economics, Nairobi, Kenya; 5 Mitra and Associates, Dhaka, Bangladesh; 6 Sight and Life, Kaiseraugst, Switzerland; 7 ETH Zurich, Zurich, Switzerland; 8 Syngenta Foundation for Sustainable Agriculture, Basel, Switzerland

**Keywords:** Secondary city, Nutrition, Stunting, Overweight, Food security, Dietary diversity

## Abstract

**Background:**

By 2050, approximately 68% of the global population will live in cities, but nutrition data on urban populations of low- and middle-income countries are scarce. Fast growing secondary cities, combining characteristics and hurdles of urban and rural settings, are hotspots for the double burden of malnutrition. The Nutrition in City Ecosystems (NICE) project focuses on 6 secondary cities in Bangladesh, Kenya and Rwanda, to improve health and nutrition, and reduce poverty. To assess the baseline situation and guide future interventions, food insecurity, dietary diversity, nutrition status, and food production and purchasing patterns were explored.

**Methods:**

In a cross-sectional study design, data were collected from urban and peri-urban households of Dinajpur and Rangpur in Bangladesh, Bungoma and Busia in Kenya, and Rubavu and Rusizi in Rwanda. Approximately 1200 households, in neighborhoods prone to malnutrition, were involved from April to June 2021. We assessed Household Food Insecurity Access Score (HFIAS), both current and before COVID-19, Household Dietary Diversity Score (HDDS), Minimum Dietary Diversity for Women (MDD-W), anthropometric measurements, household and socioeconomic information, and questions related to food production and consumer behavior. Further we collected secondary data on low birthweight and anemia during pregnancy.

**Results:**

All cities experienced a substantial increase in food insecurity during the COVID-19 pandemic. Stunting rates in children under 5 years varied among the cities and ranged from 7.8% in Busia to 46.6% in Rubavu, while half of adult women were overweight (between 42.1% in Rusizi and 55.8% in Bungoma). Furthermore, many women did not consume an adequately diverse diet (MDD-W < 5 for 29.3% in Bangladesh, 47.5% in Kenya, and 67.0% in Rwanda), however many of the urban and peri-urban households were engaged in farming (58–78%).

**Conclusions:**

The double burden of malnutrition is high in secondary cities and the COVID-19 pandemic has exacerbated levels of food insecurity. Demand for, and access to, an affordable healthy diverse diet that comprises local, nutritious, and agroecologically produced foods present a pathway for overcoming the complex challenges of malnutrition.

## Background

Even before the corona virus disease 2019 (COVID-19) pandemic, the world was facing a global nutrition crisis. The Food and Agriculture Organization (FAO) of the United Nations’ 15^th^ report of the High Level Panel of Experts (HLPE) on Food Security and Nutrition in 2020 emphasized that the world is not on track to achieve ‘Zero Hunger’ (Sustainable Development Goal 2) by 2030 [1]. Suboptimal diets are responsible for one third of the world’s population suffering from malnutrition and one fifth of adult deaths, making it the number one risk factor for disease and early death [2]. More than 720 million people suffer from hunger, 149 million children under 5 years are stunted, and over 2.3 billion people are not having regular access to sufficient, safe, and nutritious food [3, 4]. At the same time, overweight and obesity among adults, adolescents and children are rising globally. Data from 2016 reported that 131 million children aged 5–9 years and 207 million adolescents were overweight or obese [5]. Similarly, the Global Nutrition Report (GNR) 2021 indexed 38.9 million children under 5 years and 2.2 billion adults as overweight or obese [3]. Many low- and middle-income countries (LMICs) experience a double burden of malnutrition, a co-existence of over- and undernutrition [6]. Apart from restrictions in availability and accessibility of healthy and nutritious foods, the burden of infectious and chronic diseases aggravates the burden of malnutrition. Inflammation, be it chronic as for example in obese individuals or acute as during episodes of infections, can reduce the absorption of important nutrients leading to micronutrient deficiencies and result in malnutrition [7, 8]. Furthermore, exclusive breastfeeding until 6 months of age is important to ensure adequate supply of nutrients and contributes to optimal growth and development, lowers the risk for overweight and diabetes, and protects the child from infections [9].

According to the United Nations, 68% of the world’s population will live in urban areas by 2050, and around 90% of the increase in urban population will occur in small cities and towns of Africa and Asia [10]. Food and nutrition security is essential for an increase in productivity and decrease in inequalities of cities [11], but the International Food Policy Research Institute (IFPRI) estimates Asia and Africa to lose 11% of their gross domestic product (GDP) due to malnutrition each year [12]. Urban poor households in low- and middle-income countries (LMICs) tend to spend a large part of their income (up to 70%) on food, making them particularly vulnerable to food price fluctuations [13–15]. By forcing households to substitute nutritious fresh foods, such as fruits and vegetables, with less nutritious but cheaper staples, food price volatility immediately affects diet quality [16]. Furthermore, urbanization has profound effects on eating patterns [17, 18]. People relocating to urban areas experience a pronounced shift away from traditional staples and towards the consumption easily prepared foods such as rice, bread and meals away from home [17, 19–22], as well as foods that contain high levels of sugar and highly processed foods [23, 24].

The COVID-19 pandemic increased food insecurity in LMICs [25]. The long-term impact on access to and affordability of nutrient-rich foods and healthy diets will be most detrimental for vulnerable groups such as women and children, and individuals with low socio-economic status with limited resources, such as homeless people and elderly populations [26]. A systematic review by Picchioni et al*.* advocates for improved data collection to identify vulnerable groups and eventually monitor and ensure progress in reaching goals of specific programs and interventions among them [27].

To improve urban diets and support city food systems transformation towards more sustainable ways of producing and consuming food, the Swiss Agency for Development and Cooperation (SDC) initiated the Nutrition in City Ecosystems (NICE) project. By ensuring the demand and supply of diverse and nutritious foods, NICE seeks to make city food systems more nutrition-focused so they contribute to better health and nutrition status, especially among the vulnerable populations. A protocol paper by Speich et al*.* describes the NICE project in detail [28]. This study presents baseline data from vulnerable groups on i) prevalence of malnutrition indicators, ii) household and women dietary diversity, iii) relevant consumer behaviors, iv) household food insecurity access pre- and during the COVID-19 pandemic, as well as v) prevalence of low birth weight of infants and anemia among pregnant women in the six cities.

## Methods

To assess the nutritional status of the inhabitants of the secondary cities before the start of the NICE project, as well as to track progress throughout the project, we collected baseline data for each city. A cross-sectional study design using a mixed-methods approach was applied, including i) a household questionnaire (including questions on socioeconomic situation, as well as food production, purchase, preparation and consumption), ii) quantitative food consumption questionnaire specifically for women of reproductive age (WRA, 15–49 years), iii) anthropometric measurements, and iv) review of health records data from the six secondary cities.

### Study sites and data collections

**Fig. 1:**
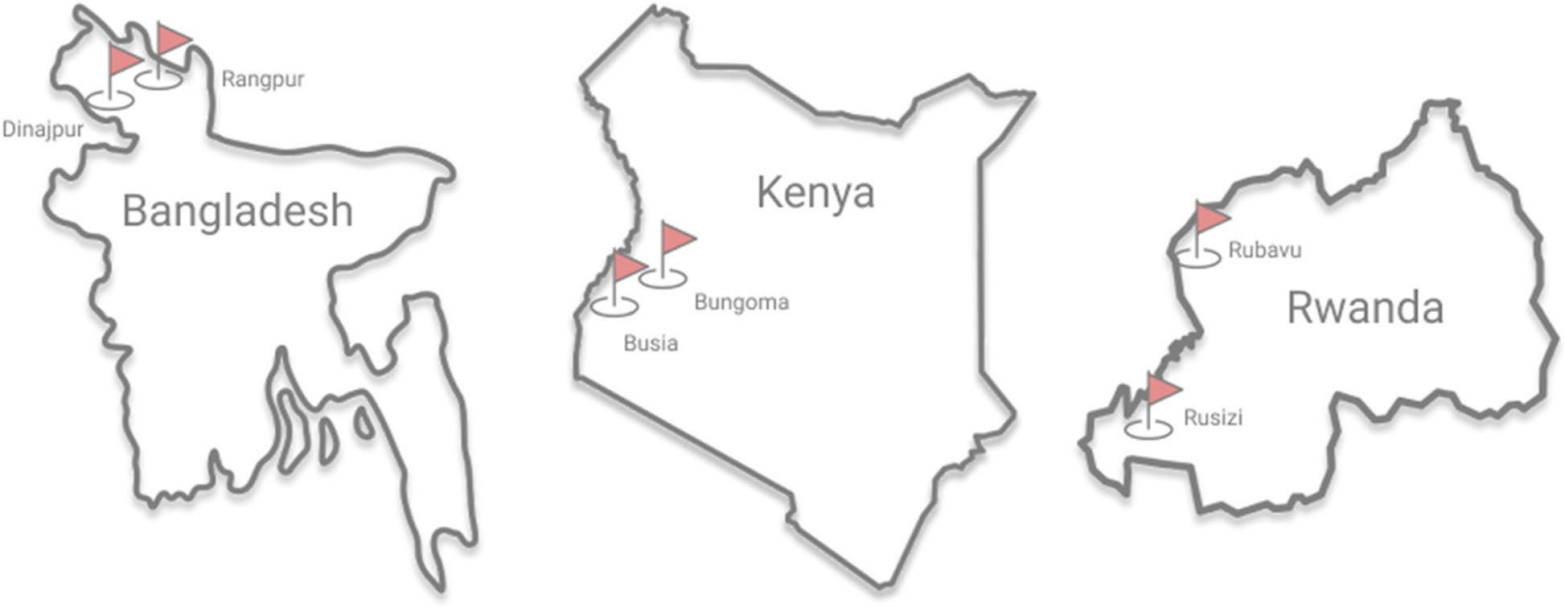
Maps of Bangladesh, Kenya, and Rwanda, indicating the location of the six secondary cities embedded in the NICE study

This study was conducted in six secondary cities in Bangladesh (Dinajpur and Rangpur), Kenya (Bungoma and Busia), and Rwanda (Rubavu and Rusizi). Study sites, and the NICE study in its whole, are described in the protocol paper by Speich et al. and shown in Fig. 1 [28].

In close collaboration with local authorities, geographical areas of the cities with known malnutrition burden were targeted (see sampling strategy). Written informed consent was obtained from all study participants (household heads, interviewed women, adult legal guardians of children and adolescents < 18 years) before participating in the study. As the interviews were conducted during the COVID-19 pandemic and thus to minimize risk for infection, all interviewers were wearing face-masks and using hand sanitizers, the same was offered to the study participants.

The household survey was administered by appropriately qualified, experienced, and carefully trained enumerators who conducted face-to-face interviews with the household member mainly responsible for food consumption in the household. The household survey questionnaire collected various forms of data, including food production practices, asset ownership, sources of income, as well as food consumption expenditure and behavior. The questionnaire also contained questions to assess the Household Food Insecurity Access Score (HFIAS) [29], a 24-hour dietary recall for food consumed in the household (Household Dietary Diversity Score (HDDS)) [30], and questions on child feeding (including breastfeeding and complementary feeding). Furthermore a 24-hour dietary recall was specifically administered to one WRA per household (using the country-adapted versions of the Diet Quality Questionnaire (DQ-Q, through direct contact with the Global Diet Quality Project group)) [31], that can be converted into a Minimum Dietary Diversity for Women (MDD-W)) [32]. In each household, height and weight were collected from all household members present and providing informed consent at the time of the visit (at least from one WRA (15–49 years) as well as either one adolescent (10–19 years) or youth (15–24 years) and/or one child under 5 years). Body weight and height, in light clothes and without shoes, were measured in kilograms (kg) and centimeters (cm) using an electronic scale and a portable stadiometer. All data collection instruments were pre-tested before the study. Data were collected electronically on android tablets using Open Data Kit (ODK). Data flow was automatized (skip patterns) and constraints set to data entry fields, to increase quality assurance. Furthermore, regular data checks were conducted by the supervisors prior to uploading the data. Data collection took place between April and May 2021 in Kenya, between May and June 2021 in Rwanda, and in June 2021 in Bangladesh.

Hemoglobin concentrations in pregnant women and birthweight of infants were collected from antenatal care (ANC) clinics (n) of the urban and peri-urban areas of Dinajpur (7), Rangpur (4), Bungoma (1), Busia (3), Rubavu (3), and Rusizi (3). Hemoglobin and birthweight data from the respective records (ANC and Growth monitoring and promotion (GMP) visits, or birth records) were collected for the period between January to May 2021 in Bangladesh and for January to March 2021 in Kenya and Rwanda, respectively.

### Sampling strategy and statistical analysis

Based on time, budget, and previous knowledge on the prevalence of key nutrition indicators, a convenient sample size of around 300 households per city in Bangladesh and 150 households per city in Kenya and Rwanda, was persued.

A two-stage sampling design was used to reach the total sample. In the first stage, informed by the fact that different malnutrition problems in urban areas tended to be clustered by residential neighborhoods, all residential neighborhoods (residential estates) in each town were listed. Whenever possible, and with the help of district local authorities, sectors with high rates of malnutrition in particular child stunting and adolescent/youth overweight and obesity were identified. The rationale to focus on these specific target group was based on the fact that these groups would be the key beneficiaries of the NICE project. We identified 10 sectors each for Dinajpur and Rangpur, 1 sector for Bungoma, 3 sectors for Busia, and 3 sectors each for Rubavu and Rusizi. Within each of the selected sectors, systematic random sampling was then used to select households. Depending on the number of sectors, the sample size of 300/150 was divided by the number of sectors and a random starting point/household within a sector was chosen. A household was included in the study if it had at least one WRA and either one adolescent (10–19 year) or youth (15–24 years) and/or one child under 5 years present. If that household met the inclusion criteria, the determined interval (interval = total number of households in that estate divided by target sample size for that estate), was used to get to the next household. If a certain household did not meet the inclusion criteria, the immediate neighboring household was approached, and the interval used again to identify the next household to screen. The exercise was stopped when the target number of households for a certain estate had been reached.

Anthropometrical measurements (weight and height) were used to calculate body mass index (BMI) for individuals aged 19 years and above with the standard formula (BMI = kg/m^2^). For children and youth under the age of 19 years, sex-specific z-scores for height-for-age (HAZ), weight-for-age (WAZ), BMI-for-age, and weight-for-height (WHZ) were calculated using the new reference equations recommended by WHO [33, 34]. These equations take into account the skewness of the respective distributions. Z-scores of -1.96 and 1.96 correspond to the 2.5^th^ and the 97.5^th^ percentiles, respectively. Stunting was defined a HAZ < -2, wasting WAZ < -2, and underweight as WHZ < -2. For participants under 19, overweight was defined as BMI-for-age z-score > 2. For participants aged 19 or older, overweight was defined as BMI ≥ 25.

The HDDS was calculated from the number of food groups consumed by any family member in the household within the previous 24 hours. The 12 groups were: 1-cereals; 2-roots and tubers; 3-vegetables; 4-fruits; 5-meat, poultry, offal; 6-eggs; 7-fish and seafood; 8-pulses, legumes, nuts; 9-milk and milk products; 10-oil/fats; 11-sugar/honey; 12-miscellaneous. Food consumed outside of the household were not considered [35]. The MDD-W was calculated from the number of food groups consumed by WRA in the past 24 hours. The following 10 groups were considered for MDD-W: 1-grains, plantains, and white roots and tubers; 2-pulses like beans, peas and lentils; 3-nuts and seed; 4-dairy; 5-meat, poultry and fish; 6-eggs; 7-dark green leafy vegetables; 8-other vitamin A-rich fruits or vegetables; 9-other vegetables; 10-other fruits [36]. The percentage of WRA consuming less than 5 food groups was calculated, indicating an insufficiently diverse diet. Furthermore, household food insecurity access was assessed using a set of questions on the lack of sufficient and adequate food, using the HFIAS approach [37]. We used the HFIAS to access present situation as well as the situation prior to the COVID-19 pandemic. The share of food expenditure was used to get an insight into the percentage of money spent on food from the total weekly household expenditure and salary. Further we assessed which local or imported foods were regularly consumed. In addition, the percentage of women taking supplements and infants receiving and consuming fortified porridge was calculated. The prevalence of anemia in pregnant women was calculated based on secondary data accessed via antenatal care clinics. The WHO definition of anemia in pregnancy was used with hemoglobin concentration of less than 11 g/dl [38]. Low birth weight was defined using the WHO cut-off of < 2500 g [39].

Data cleaning and analysis was done using STATA IC16. All data were checked for completeness and outliers and descriptive statistics were used to provide age- and gender-stratified prevalence and means or medians.

## Results

Overall, we interviewed and included in our analysis 307 households in Dinajpur, 299 in Rangpur, 151 in Busia, 150 in Bungoma, 150 in Rubavu, and 150 in Rusizi. The majority of households reported a male household head, namely 89.9% in Dinajpur, 93.3% in Rangpur, 82.0% in Bungoma, 78.9% in Busia, 84.0% in Rubavu, and 80.0% in Rusizi. The gender, average age, and level of education (no school, and primary, secondary, and tertiary education) of the household head in each city population are shown in Table 1.

**Table 1:**
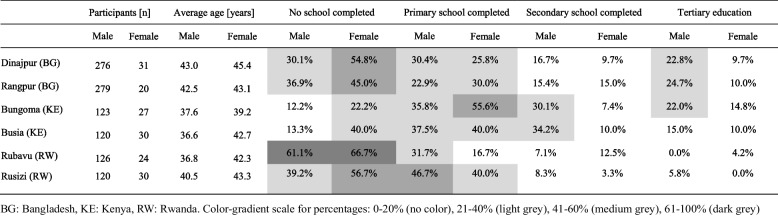
Education status among household heads in NICE cities

### Agriculture, livestock and water

**Fig. 2:**
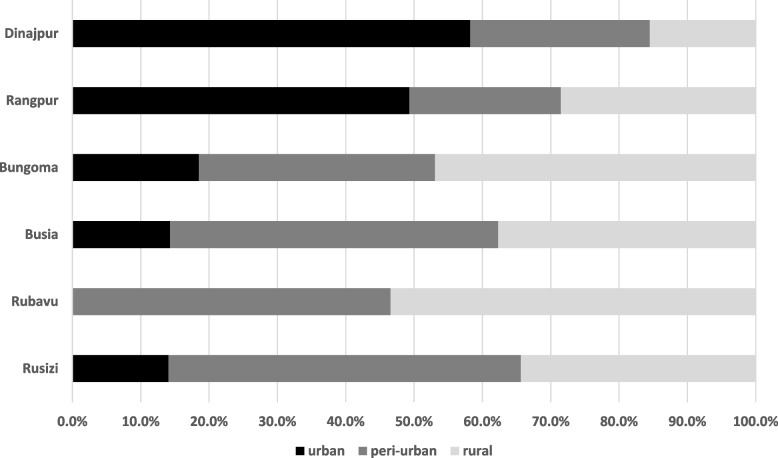
Location (urban, peri-urban or rural) of farmland owned or accessed by the interviewed households of the NICE cities (Bangladesh (Rangpur and Dinajpur), Kenya (Bungoma and Busia), and Rwanda (Rubavu and Rusizi))

Farming activities were assessed through the owning or accessing of agricultural land. Important to note, most of the participants reported an additional occupation and source of income besides these agricultural activities. However, in Bangladesh, 69.4% of the households in Dinajpur were involved in farming, 77.9% of them had less than 12 acres of farmland (smallholder farm) and 23.9% of them had 2 acres or less (marginal farm). In Rangpur 74.3% were involved in farming, 70.6% of them had less than 12 acres and 22.1% had 2 acres or less. In Kenya, 63.3% of the households in Bungoma and 66.0% in Busia were involved in farming; 100% in both cities had smallholder farms of which 86.4% and 88.3% were marginal farms, respectively. In Rwanda, 58.0% of the households in Rubavu and 59.3% in Rusizi were involved in farming; 31.5% and 25.4% in smallholder farms whereof 16.6% and 20.6% were marginal farms, respectively. The farmland in the Bangladeshi cities was mainly located in urban areas, in the African cities the majority was in the peri-urban or rural areas (Fig. 2). Only few of the responding households had received loans or support for agriculture (cash or in kind) during the past year; for Bangladesh, 1.3% in Dinajpur and Rangpur each; for Kenya, 4.0% in Bungoma, 6.0% in Busia; and for Rwanda, 5.3% in Rubavu and 14.7% in Rusizi, mostly from NGOs, funds or government sources.

For Bangladesh, in Dinajpur, 30.9% of the households possessed livestock, mainly chicken (17.3%), goats (12.7%), cattle (8.8%), and ducks (5.5%). In Rangpur, 26.8% of the households had livestock, mainly chicken (11.0%), goats (8.4%), ducks (6.7%), and cattle (4.3%). For Kenya, in Bungoma, 57.3% of the households possessed livestock, mainly chicken (56.0%), cattle (17.3%), goats (11.3%), and pigs (4.7%). In Busia, 58.0% of the households had animals, mainly chicken (34.7%), cattle (19.3%), goats (12.7%), and pigs (3.3%). For Rwanda, in Rubavu, 35.3% of the households possessed livestock, mainly chicken (13.3%), cattle (10.7%), pigs (10.7%), goats (6.0%), sheep (2.7%), and rabbits (2.0%). In Rusizi, 50.0% of the households had animals, mainly pigs (23.3%), cattle (21.3%), chicken (16.0%), goats (8.7%), sheep (5.3%), and rabbits (4.0%).

In Bangladesh, most households accessed a bore hole to source their drinking water, whereas in Kenya and Rwanda more often protected or unprotected well or springs, public standpipes, piped into a dwelling or plot, and surface water were used (see Table 2). Furthermore, for Bangladesh the majority of households had a flush toilet, in Dinajpur 45.9% had a flush toilet to a septic tank, 37.8% to a pit and 1.3% to a piped sewage system; another 3.9% used a traditional pit latrine and 11.1% a ventilated improved pit latrine (VIP). In Rangpur, 40.1% had a flush toilet to a septic tank, 45.2% to a pit and 4.0% to a piped sewage system; another 1.7% used a traditional pit latrine and 8.4% a VIP, 0.7% had no facility. For Kenya, in Bungoma 40.0% of the households used a traditional pit latrine, 35.3% a VIP, 10.0% a flush toilet to a piped sewing system, 10.0% a flush toilet to a septic tank, and 4.0% a flush toilet to a pit. In Busia, 39.3% of the households used a traditional pit latrine, 42.0% a VIP, 8.7% a flush toilet to piped sewing system, 8.0% a flush toilet to septic tank, and 1.3% a flush toilet to a pit. For Rwanda, in Rubavu the vast majority of households used a traditional pit latrine (81.3%), some a flush toilet to pit (13.3%), 3.3% had no facility and 2.0% use the neighboring facility. In Rusizi, 83.3% owned a pit latrine, 16.0% a flush toilet to a pit, and 0.7% had no facility.

**Table 2:** Sources of drinking water in the households of the NICE cities

	Bore hole	Protected well/spring	Public tap/standpipe	Piped into dwelling/plot	Surface water	Bottled water	Unprotected well/spring
Dinajpur (BG)	98.0%	0.0%	0.0%	2.0%	0.0%	0.0%	0.0%
Rangpur (BG)	96.3%	0.3%	0.3%	3.0%	0.0%	0.0%	0.0%
Bungoma (KE)	16.7%	24.7%	27.3%	22.7%	6.7%	0.7%	1.3%
Busia (KE)	8.0%	42.7%	10.7%	22.7%	10.7%	0.7%	4.0%
Rubavu (RW)	0.0%	4.7%	71.3%	12.0%	4.7%	0.0%	7.3%
Rusizi (RW)	0.0%	24.7%	18.0%	24.7%	8.0%	2.7%	22.0%

One household in Busia reported to use rainwater

*BG* Bangladesh, *KE* Kenya, *RW* Rwanda

### Food consumption and purchasing patterns

Many households were food insecure, defined as not being able to eat the desired variety, quality or quantity of foods. The indication for food insecurity (assessed by HFIAS) substantially increased during the COVID-19 pandemic in all cities. In Dinajpur from 34.9% before the pandemic to 54.1% during the pandemic, in Rangpur from 33.4% to 55.2%, in Bungoma from 77.3% to 88.7%, in Busia from 76.0% to 88.7%, in Rubavu from 81.3% to 98.7%, and in Rusizi from 86.0% to 99.3%. Median (interquartile range (IQR)) weekly household expenditure from salary for food during the pandemic varied from 36.9% (30.0–50.1) in Rangpur to 65.2% (37.0–100.0) in Rusizi, see Table 3. Especially in Rwanda, several households reported to spend all their weekly income on food, although not spending any money for some food groups such as dairy or also meat and fish. Overall, most people indicated to consume mainly local food. In Bangladesh the purchase of imported fruits and legumes was common, also the Kenyan cities Busia and to some extent Bungoma reported to consume some substantial amounts of imported foods (Table 4).

**Table 3:** Median (interquartile range) weekly household expenditures for food as percentage of weekly salary expended for different food groups per week

	Total expenditures for food	Expenditures for fruits	Expenditures for vegetables	Expenditures for dairy	Expenditures for protein legumes	Expenditures for meat	Expenditures for fish
Dinajpur (BG)	39.7% (30.0–50.1)	14.3% (11.1–18.8)	20.1% (15.6–26.0)	8.1% (5.6–11.5)	3.9% (2.6–6.3)	25.7% (20.3–62.2)	22.6% (19.0–52.1)
Rangpur (BG)	36.9% (28.1–47.8)	15.3% (11.3–22.0)	19.2% (15.2–24.8)	8.1% (4.9–11.7)	3.2% (2.2–4.5)	27.3% (21.6–33.5)	22.4% (18.5–26.3)
Bungoma (KE)	47.6% (26.8–79.9)	11.1% (5.7–16.7)	17.8% (10.2–27.0)	24.6% (15.2–31.4)	6.8% (3.9–11.8)	15.9% (10.0–22.4)	15.1% (8.8–20.8)
Busia (KE)	50.8% (33.8–92.7)	10.6% (5.6–15.8)	17.4% (10.5–25.9)	20.5% (12.3–28.4)	6.9% (4.2–12.0)	16.4% (10.5–25.4)	18.2% (12.5–26.6)
Rubavu (RW)	58.7% (34.8–100)	5.9% (0–13.8)	14.3% (4.8–22.2)	0.0% (0–7.4)	47.6% (28.6–63.6)	0.0% (0–18.5)	10.2% (0–20.5)
Rusizi (RW)	65.2% (37.0–100)	11.4% (0–18.8)	17.0% (9.1–31.3)	0.0% (0–10.3)	22.2% (12.0–45.5)	3.7% (0–25.0)	13.7% (3.9–25.0)

*BG* Bangladesh, *KE* Kenya, *RW* Rwanda

**Table 4:**
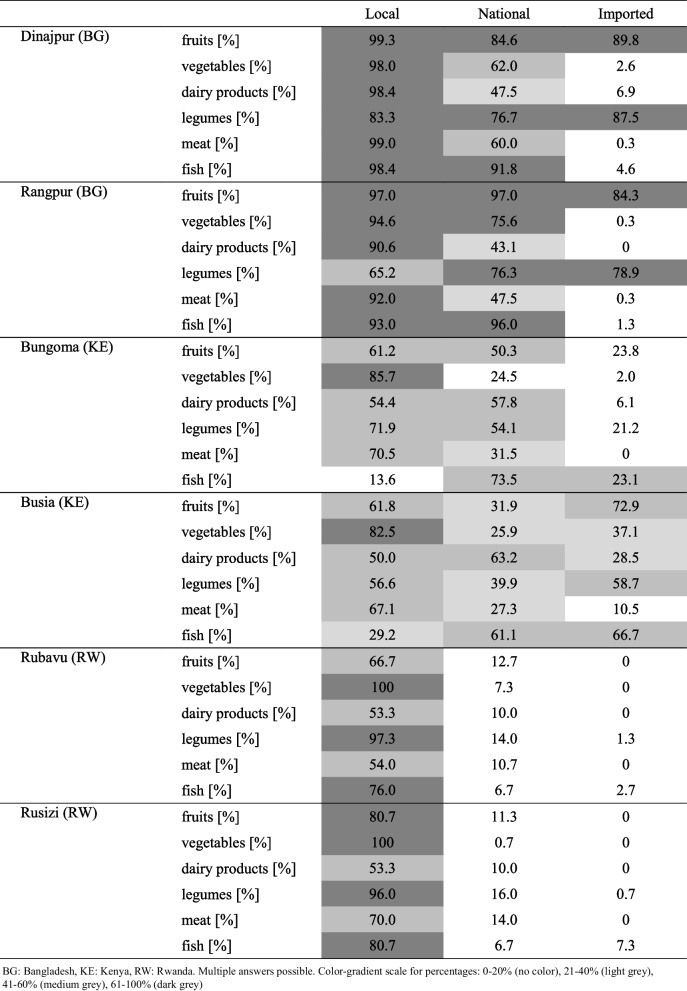
Provenance of food as percentage of food of local, national and imported provenance in NICE cities

**Fig. 3:**
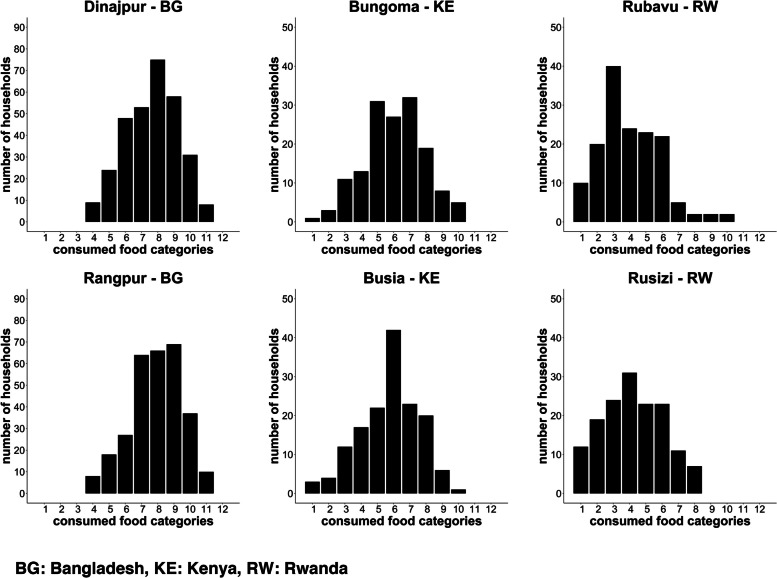
Household Dietary Diversity Score in the NICE cities

The Household Dietary Diversity Score (HDDS) varied from the consumption of an average of 4.0 ± 1.9 out of 12 food categories in Rubavu (Rwanda), to 7.9 ± 1.6 food categories consumed in Rangpur (Bangladesh, Fig. 3). Households lead by a male household head in Kenya and Rwanda had the tendency of a higher mean HDDS (6.0 and 4.1) compared to households with a female head (5.3 and 3.8). In Bangladesh, households with a female household head tended to have a higher HDDS compared to male headed households (8.1 compared to 7.7). In Rwanda few households consumed processed foods at home in the previous 24 h the least frequent (8.0% in Rubavu and 16.0% in Rusizi), on the other side in Kenya and Bangladesh more than half of the households reported the consumption of processed foods at home (58.9% in Busia, 57.3% in Bungoma, 61.0%in Dinajpur, and 71.8% in Rangpur).

**Fig. 4:**
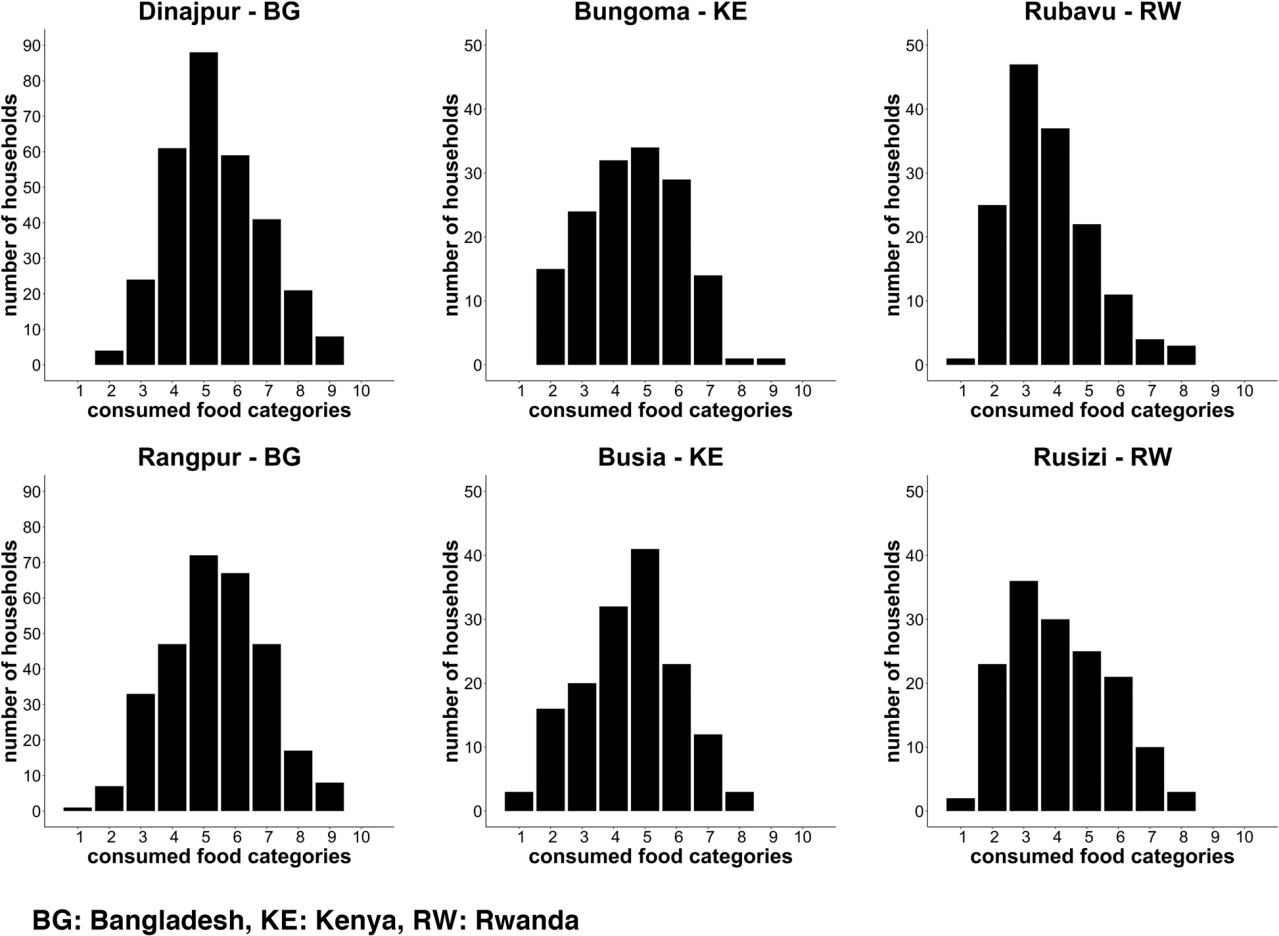
Minimum Dietary Diversity for Women score calculated for responding WRA in the NICE cities

The Minimum Dietary Diversity for Women (MDD-W) score varied from the consumption of a mean of 3.8 ± 1.4 out of 10 food categories in Rubavu to a mean of 5.4 ± 1.6 food categories consumed the previous 24 h in both, Dinajpur and Rangpur (Fig. 4). A balanced diet, defined as the consumption of 5 or more food groups, was reported by 70.9% of the WRA in Dinajpur, 70.6% in Rangpur, 52.7% in Bungoma, 52.3% in Busia, 26.7% in Rubavu, and 39.3% Rusizi.

In Bangladesh, only few WRA reported to consume supplements (7.8% in Dinajpur and 6.4% in Rangpur) and even less common was fortified weaning food such as porridge (0.6% in Dinajpur and 0.7% in Rangpur). In Kenya, one in four WRA took supplements (28.7% in Bungoma and 23.2% in Busia, containing all kind of micronutrients such as iron, zinc, folic acid, vitamin A, C, D, and multivitamins) and 34.7% in Bungoma and 25.2% in Busia gave fortified porridge to the children. In Rwanda, very few WRA reported to take supplements (5.3% in Rubavu and 2.0% in Rusizi), but 23.3% and 21.3% of the children received fortified porridge (Shisha Kibondo) in Rusizi and Rubavu, respectively.

### Anthropometric measurements

Prevalence of stunting, wasting, under- and overweight in children under 5 years, are presented in Table 5. The highest rates of stunting were found in Rwanda, with almost half of the children in Rubavu being stunted and over a quarter of the children in Rusizi being too short for their age, followed by the cities in Bangladesh (Rangpur 22.0% and Dinajpur 15.7%), and the lowest prevalence of stunting in Kenya (Bungoma 12.6% and Busia 7.8%). Wasting and underweight on the other hand were lowest in Rwanda compared to Kenya and Bangladesh, where it affected up to one in seven or eight children. Overweight was overall quite low in children under 5 years. Highest prevalence was found in Rwanda, where around 10% were affected.

**Table 5:** Status of malnutrition in children under 5 years in the NICE cities

	n	Stunting	Wasting	Underweight	Overweight
Dinajpur (BG)	305	15.7%	15.4%	12.5%	3.0%
Rangpur (BG)	273	22.0%	11.0%	7.7%	4.0%
Bungoma (KE)	159	12.6%	8.8%	8.2%	7.5%
Busia (KE)	179	7.8%	13.4%	12.8%	4.5%
Rubavu (RW)	178	46.6%	3.4%	2.8%	10.1%
Rusizi (RW)	191	28.3%	2.1%	2.1%	11.0%

Stunting defined as height-for-age Z-score < -2, wasting defined as weight-for-age Z-score < -2, underweight defined as weight-for-height Z-score < -2 and overweight defined as BMI-for-age Z-score > 2

*BG* Bangladesh, *KE* Kenya, *RW* Rwanda

The rates of overweight and obesity among adolescents, youth, and adults are shown in Table 6. In Bangladesh, the prevalence of overweight and obesity was already visible in adolescents (6.0–8.0%), whereas this was rare in the African cities (0.0%-2.9%). Overweight and obesity rates in youth were higher, especially in the female population where they ranged between 12.0% in Rubavu and 18.8% in Dinajpur. For adults, again women were showing higher prevalence of overweight and overall, almost 50% of the WRA were overweight (ranging from 42.1% in Rusizi to 55.8% in Bungoma). Overweight in men was less frequent and much less homogeneous across the cities (ranging from 6.9% in Rubavu to 42.6% in Dinajpur).

**Table 6:** Prevalence of overweight and obesity among adolescents, youth, and adults

	Adolescents (10–19 years)		Youth (15–24 years)		Adults (19–49 years)	
	Female (n)	Male (n)	Female (n)	Male (n)	Female (n)	Male (n)
Dinajpur (BG)	6.0% (184)	8.0% (113)	18.8% (112)	7.7% (26)	53.4% (350)	42.6% (61)
Rangpur (BG)	6.8% (162)	7.0% (143)	17.6% (102)	1.9% (53)	45.5% (341)	26.9% (78)
Bungoma (KE)	1.5% (66)	0.0% (39)	14.8% (61)	10.5% (19)	55.8% (154)	18.2% (11)
Busia (KE)	1.4% (72)	2.9% (35)	15.9% (69)	0.0% (10)	53.5% (155)	30.0% (10)
Rubavu (RW)	0.0% (30)	0.0% (21)	12.0% (25)	0.0% (5)	44.8% (143)	6.9% (29)
Rusizi (RW)	2.3% (43)	0.0% (30)	14.7% (34)	0.0% (11)	42.1% (145)	29.0% (31)

Overweight and obesity defined as BMI ≥ 25 kg/m^2^ in individuals aged 19 years or more and a BMI-for-age Z-score > 2 in individuals under 19 years

*BG* Bangladesh, *KE* Kenya, *RW *Rwanda

### Antenatal care data and exclusive breastfeeding

While anemia prevalence among pregnant women varied between 9.9% in Rangpur and 30.0% in Bungoma (no prevalence calculation could be done for Rwanda due to the fact that generally no records are kept for women tested negative for anemia), percentage of low birth weight children (< 2500 g) varied from 6.3% in Bungoma to 19.6% in Rangpur (also here, no prevalence calculation could be done for Rwanda due to the same methodological issue; Table 7).

**Table 7:** Exclusive breastfeeding during the first 6 months of life, low birth weight, and anemia prevalence in pregnant women

	Exclusive breastfeeding during the first 6 months (n)	Low birth weight (< 2500 g) (n)	Pregnant women with anemia (< 11 g/dl) (n)
Dinajpur (BG)	59.4% (301)	18.9% (1851)	21.4% (1637)^a^
Rangpur (BG)	52.3% (298)	19.6% (1771)	9.9% (4644)^a^
Bungoma (KE)	47.0% (136)	6.3% (3077)	30.0% (3112)
Busia (KE)	61.9% (168)	8.1% (869)	24.1% (1230)
Rubavu (RW)	62.6% (169)	-	-
Rusizi (RW)	50.0% (181)	-	-

No low birth weight and anemia assessment possible in Rwanda as no records kept for total number of deliveries and total number of pregnant women not suffering from anemia as no records kept for such cases

Exclusive breastfeeding data collected during household visit within this survey. Low birth weight data and anemia prevalence data collected through review of respective ANC records from Jan-May2021 in Bangladesh and from Jan-Mar2021 in Kenya and Rwanda

*BG* Bangladesh, *KE* Kenya, *RW* Rwanda

^a^only for NGOs’ antenatal care centers as governmental hospitals/clinics don’t keep records of hemoglobin/anemia

During the household visits we asked about the breastfeeding of all children under 5 years of responding WRA. Only about half of all children from the visited households have been exclusively breastfed until 6 months of age (ranging from 47.0% in Bungoma to 62.6% in Rubavu (Table 7).

## Discussion

Research on secondary cites has been limited, especially nutrition and food systems research; although the majority of the world’s urban dwellers live in secondary cities [16, 40]. This study disseminates important nutrition-related data collected in vulnerable populations of secondary cities in Bangladesh, Kenya, and Rwanda. Acting as “nodal points between the rural and the urban” [40], households in secondary cities were still mainly traditionally organized, with men heading the majority of them. Furthermore, despite living in urban and peri-urban city regions, still more than half of the people were engaged in farming, with generally very small plots of accessible farmland that were situated inside the city (urban, peri-urban) but also in its rural surrounding.

Urbanization has been shown to be associated with a change in diets from traditional fresh foods to more pre-prepared and processed foods along with sugar sweetened beverages, containing more fat, sugar, and salt [41–44]. Our results confirmed high consumption of processed foods, especially in the Bangladeshi and Kenyan cities, where more than half of all visited households consumed processed foods the previous 24 hours. This nutrition transition shows its effects, across all secondary cities in our study, 42.1–55.8% of the WRA and 6.9–42.6% of men were overweight, starting already with 3.0–11.0% of children under 5 years. Popkin and Slining reported 32% of the urban population in Sub-Saharan Africa to be overweight versus 16% in rural areas, and 28% of the urban population in South Asia compared to 10% in rural areas in 2010 [45]. For Bangladesh, the Global Nutrition Report (GNR) published overweight and obesity prevalences for children under 5 years of age of 2.4% (2019), 5–19 years old boys of 9.3% (2016), 5–19 years old girls of 8.7% (2016), and WRA of 22.2% (2016) [46]. Our adolescent study population (10–19 years) in Bangladesh had a similar prevalence (6.0–6.8% in girls and 7.0–8.0% in boys). The last Demographic Health Survey (DHS) (2017–18) reported 43.4% of all ever-married urban WRA of Bangladesh were overweight or obese [47], similar or slightly lower to the 45.5% (Rangpur) and 53.4% (Dinajpur) of overweight and obesity prevalence in our study. The fact that DHS published an overall overweight and obesity prevalence of only 24.3% for the whole Rangpur division, to which Dinajpur and Rangpur both belong to, underlines the higher prevalence of overweight and obesity in urban compared to rural populations [47]. For Kenya, the GNR reported national overweight and obesity prevalence for children under 5 years of age of 4.2% (2014), 5–19 years old boys of 6.2% (2016), 5–19 years old girls of 16.2% (2016), and WRA of 34.3% (2016) [48]; again lower than in children and WRA of our study. Only the adolescents in our survey had a lower prevalence in overweight (0.0–2.9%) compared to the GNR. On the other hand, older data from a Kenyan DHS in 2014, reported an overweight and obesity prevalence of 43.3% for urban women [49], this prevalence is closer to the 53.5–55.8% overweight and obesity prevalence found in our study 7 years later. In Rwanda, the GNR published national overweight and obesity prevalence for children under 5 years of age of 5.6% (2020), 5–19 years old boys of 5.2% (2016), 5–19 years old girls of 16.9% (2016) and of WRA 33.5% (2016) [50]. Overweight and obesity prevalence in Rwandan children under 5 years in our survey was with 10.1–11.0% substantially higher than in the GNR and more than twice as high as in Bangladesh and Kenya. Also, the overweight in Rwandan WRA in our study was higher (42.1–44.8%) compared to the data from the GNR. On the other hand, the Rwandan adolescents of our survey were showing almost no obesity (only girls in Rusizi, with 2.3%), but here the sample size was very low.

Despite having specifically selected neighborhoods with a known high burden of malnutrition, stunting prevalence in Bangladeshi children under 5 years was with 15.7% (Dinajpur) and 22.0% (Rangpur) lower than in the DHS 2017–18 for Rangpur division (30.4%) as well as the general urban population (25.4%) [47]. Also for Kenya, the prevalence of stunting in children under 5 years in Bungoma (12.6%) and Busia (7.8%) were substantially lower than reported 2014 nationally (26.2%) and in the urban population (20.0%) [48, 49]. In Rwanda, the 2019–2020 DHS reported a national under 5 stunting prevalence of 33.1% and in urban settings 19.8%, with substantially higher prevalence in the Western province (40.2%) [50, 51]. Among our study cities, Rubavu, in the Western province, had a particularly high stunting prevalence with almost half of all the children (46.6%) under 5 years being affected, not only compared to national data but also compared to all other involved cities. Looking at wasting in children under 5 years, figures were considerably different; while stunting prevalence was highest in Rwanda, wasting prevalence was lowest among all NICE countries and only slightly above the data from the GNR (1.1% in national and 1.6% in urban population) [50]. On the other hand, in the Bangladeshi and Kenyan cities compared to other available urban data, wasting prevalence were higher in our study population (in the Bangladeshi GNR in 2019 urban wasting prevalence was 8.7%, in the Kenyan GNR in 2014 urban wasting prevalence was 3.5%) [3, 47, 49, 51]. To summarize on undernutrition in children under 5 years, stunting as a long-term malnutrition indicator seemed to be less prominent in the study population of Kenya and Bangladesh. Interestingly however, wasting, as an immediate sign of recent poor nutrition, was much higher in all 6 study cities, compared to available national data (urban or overall). This may be a direct effect of the COVID-19 pandemic, as demonstrated recently by a study of Heady and Ruel indicating negative economic shocks substantially increasing the risk of all types of wasting [52].

Diets consumed by many WRA in our survey were rather poor, with highest percentage of insufficient diversity in Rwanda (73.3% in Rubavu, 60.7% in Rusizi) and lowest in Bangladesh (29.1% in Dinajpur, 29.4% in Rangpur). Bangladeshi households were spending a higher percentage of their food expenditures for fruits, this was to a lesser extend seen in the African cities. This could be contributing to the higher probability to consume a balanced diet based on the MDD-W score (70.7% compared to 52.5% in Kenya and 33.0% in Rwanda). Also, particularly the food consumed at home was most diverse in Bangladesh, based on the HDDS. Furthermore, in Rwanda, lowest percentage of study respondents were engaged in farming (58.0% and 59.3% in Rubavu and Rusizi, respectively) what might contribute to the poor dietary diversity. Furthermore, household expenditures (relative to the income) for food were the highest in Rwanda, where many respondents indicated to spend all their income for food. Previous studies on urban food insecurity have shown high food expenditures of the city inhabitants but our findings exceed the known previous data [13–15]. Being highly dependent on food price volatility, nearly all interviewed households (> 99%) had an indication of food insecurity based on HFIAS in Rwanda (May/June 2021) even though this percentage has already been very high in pre-COVID-19 times (> 80%). We would like to note here the potential bias of having people reporting on a situation from such a long time ago. However, percentage of households having indications of food insecurity during COVID-19 also increased in Kenya and Bangladesh. Bangladesh, in line with lowest share of food expenditures, was also having the lowest percentage of food insecure households before (~ 34%) and during the COVID-19 pandemic (~ 55%). Recent data by Egger et al. have shown as well the tremendous effect of the pandemic on income, employment and access to markets in many countries, among them Rwanda, Kenya and Bangladesh [25]. An increase in food prices and therefore food insecurity is seen globally [53].

Interestingly, food expenditures allocation to different food groups was comparable between the two secondary cities within each country, but varied widely among the countries. While Bangladeshi participants spent around a quarter of their income on meat, they only spent ~ 4% for protein legumes. In Kenya, expenditures were highest for dairy (24.6% in Bungoma, 20.5% in Busia) and lowest for protein legumes (~ 7%) while meat accounted for ~ 16% of the food expenditures. In Rwanda, in line with generally high food expenditures, protein legumes (beans being a staple in Rwanda) was the food group most money was spent on (up to 47.6% in Rubavu) and expenditures for dairy and meat were very low, indicating that many people might not have purchased these generally expensive products at all. When asking about the origin of their food, more than 96.0% of Rwandan respondents declared to purchase locally produced legumes and only 14.0% to 16.0% of respondents confirmed to also buy legumes imported from other regions or countries. Similar for fruits, where only 11.3% to 12.7% of respondents confirmed to purchase fruits from other regions (but not other countries). Overall, the purchase and consumption of imported food (nationally and internationally) was particularly low among the population of Rubavu and Rusizi. In Bangladesh on the other hand, despite the large variety of indigenous fruits and legumes, 3 out of 4 households reported to also consume imported fruits and vegetables, while the percentage of imported foods was low for the other categories such as dairy, fish, meat, and vegetables. As the number of households reporting having livestock was lowest in Bangladesh, most of these foods, would then be bought from local or regional sources. In Kenya, in Busia, directly at the Ugandan border, a lot of fish, fruits and legumes were imported while substantially less dairy, meat and vegetables were imported. In Bungoma, further away from the Ugandan border, much less food was imported, but more legumes on the markets were from local and national origin. Overall, this study shows that the majority of the food purchased by the peri-urban and urban households are produced locally around the cities. This is in line with a global analysis by Thebo et al*.* showing that most croplands (~ 60% and ~ 35% of all irrigated and rain fed croplands, respectively) are within 20 km around the cities [54], making them a semi-closed food system with a lot of potential.

According to the GNR, Bangladesh, Kenya and Rwanda are on course regarding the global nutrition target of exclusive breastfeeding until 6 months by 2025 (> 50%) [55]. Also, the data collected during the household visits in the six secondary cities displayed exclusive breastfeeding rates in the first 6 months of above 50%, with the exception of Bungoma (47.0%). When comparing the 59% exclusive breastfeeding rate of the urban population from the GNR in Bangladesh, it was similar to our study cities (Dinajpur 59.4% and Rangpur 52.3%) [56]. However, the GNR data on exclusive breastfeeding for urban Kenya (70.9%) and Rwanda (78.7%) were much higher than what the mothers reported during our household survey (Bungoma 47.0%, Busia 61.9%, Rubavu 62.6%, Rusizi 50.0%) [57, 58].

The secondary data collected on anemia prevalence varied largely between the two cities in Bangladesh (21.4% in Dinajpur and 9.9% in Rangpur) and was lower than the 42.2% presented by GNR in 2019 [46]. It needs to be kept in mind that for Bangladesh only data from NGO hospitals were taken into consideration as government hospitals did generally not keep hemoglobin records, furthermore the sample size was again limited. Also in Kenya, anemia prevalence among pregnant women was lower than the 40.3% stated in GNR for 2019 [48].

Low birthweight is an important nutrition indicating maternal and fetal health, and through the life-course predicting mortality, stunting, and adult-onset chronic conditions [59]. The secondary data of birthweight collected from the health centers in the study area in Kenya and Bangladesh for the first months of 2021 presented lower prevalence of low birthweight than reported in GNR for 2015 (Bangladesh: ~ 19% compared to 27.8% in 2015 and even 58.3% as the urban prevalence in the DHS 2017–18 [47]; Kenya: ~ 7.5% compared to 11.5% in 2015), but there have been very steady decreases in low birthweight rates since 2010 in all 3 countries according to GNR [46, 48, 50].

## Conclusions

This study presents specific and novel data on city food systems and nutrition status of inhabitants of six secondary cities from three different countries. Our data underline, that vulnerable populations of secondary cities, often forgotten in food systems research so far, show characteristics of both, rural and urban areas. A nutrition transition from traditional, more diverse, fresh, and local foods to more convenient, less nutritious, and more processed foods could be seen and may contribute to the double burden of malnutrition. At the same time indications for food insecurity are rising tremendously, especially with the current challenges and shocks such as the COVID-19 pandemic, climate change, and other socio-economic impacts. Food expenditures are generally high but, the majority purchased and consumed foods are mainly produced locally around the cities. Therefore, increased availability and accessibility to affordable, diverse, nutritious, and locally produced foods at the city markets would benefit the urban population.

## Data Availability

The datasets analysed during the current study are available from the corresponding author on reasonable request.
